# Supplementary figures and images for: Predicting Potential Distribution of Teinopalpus aureus Integrated Multiple Factors and Its Threatened Status Assessment
Source: Insects. 2024 Nov 9;15(11):879. doi: 10.3390/insects15110879 (PMC11594619; doi:10.3390/insects15110879)

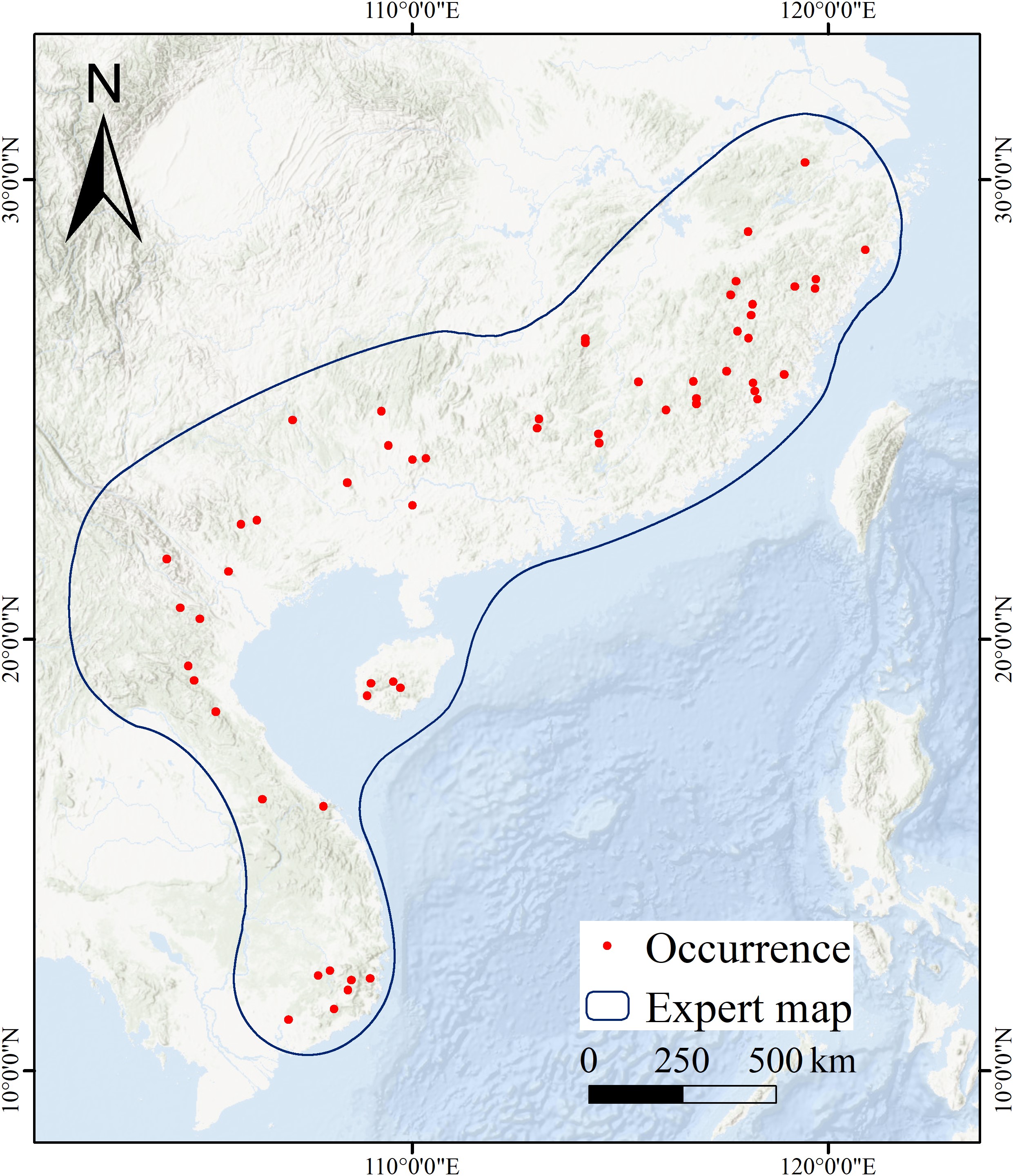

Supplement: Supplementary file 1 [file insects-15-00879-s001.zip › Fig. S1 The occurrence points and expert map of T. aureus.jpg]

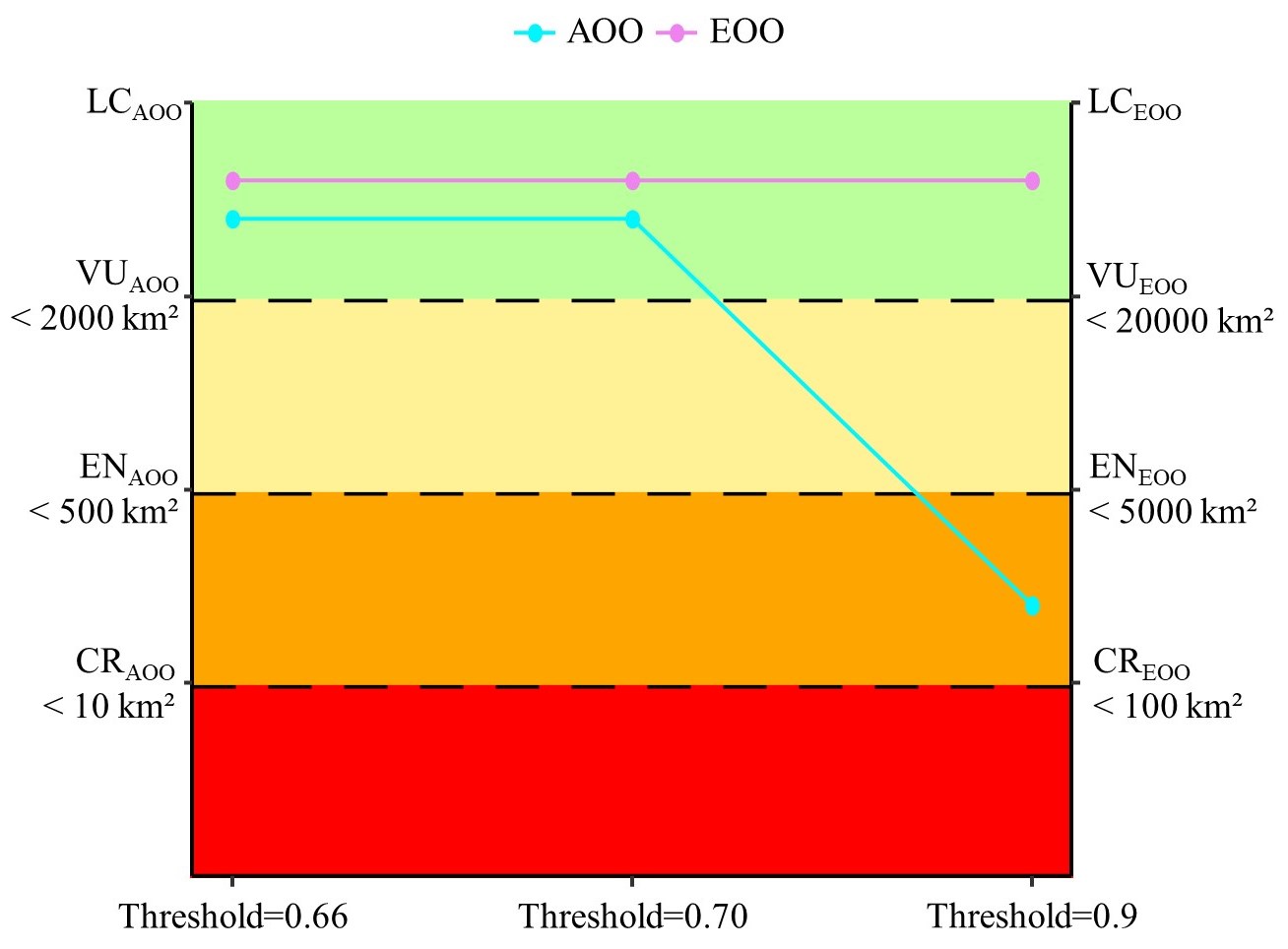

Supplement: Supplementary file 1 [file insects-15-00879-s001.zip › Fig. S10 The AOO and EOO value of T. aureus under differernt likelyhood.jpg]

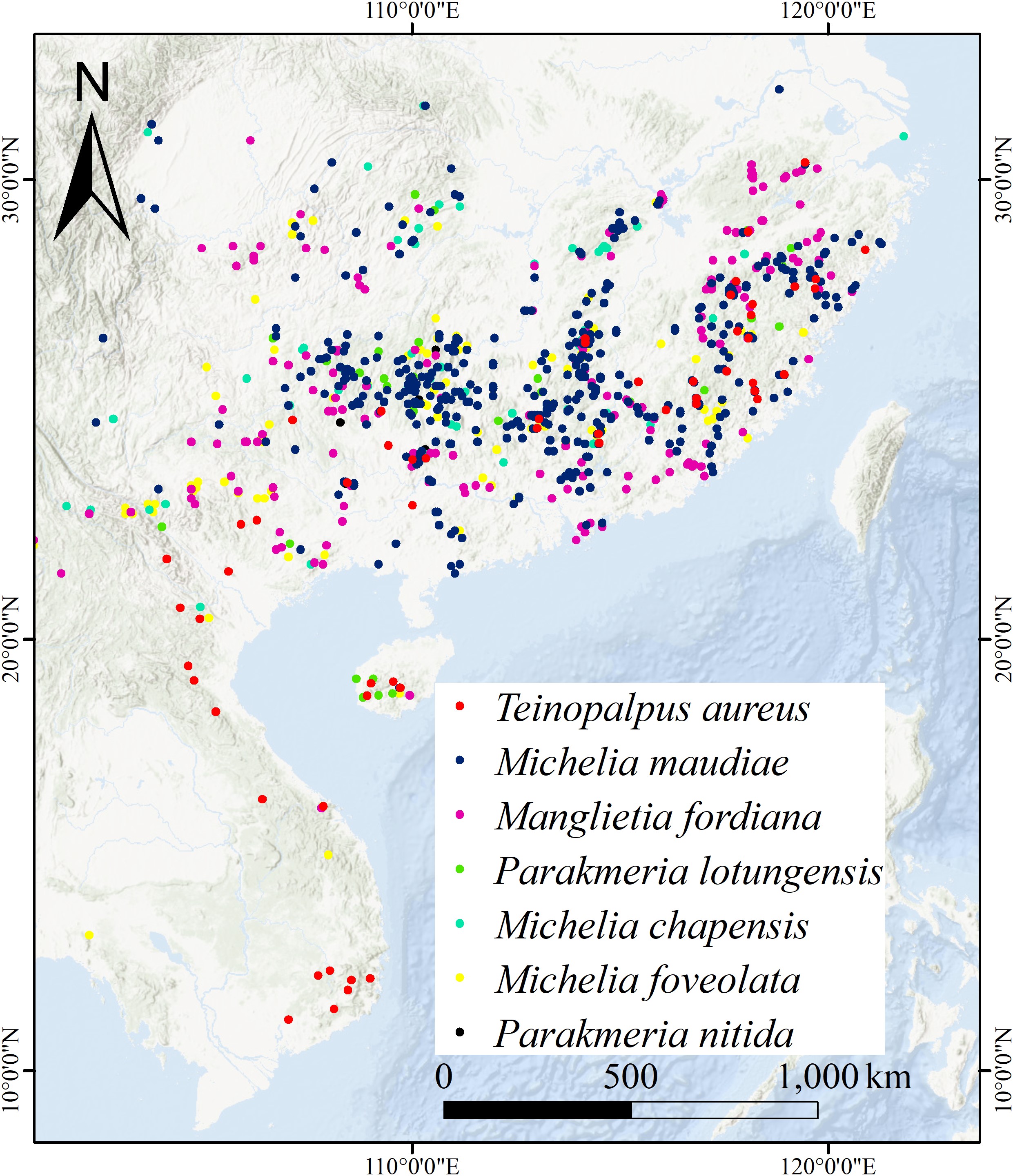

Supplement: Supplementary file 1 [file insects-15-00879-s001.zip › Fig. S2 The occurrence points of six host plants.jpg]

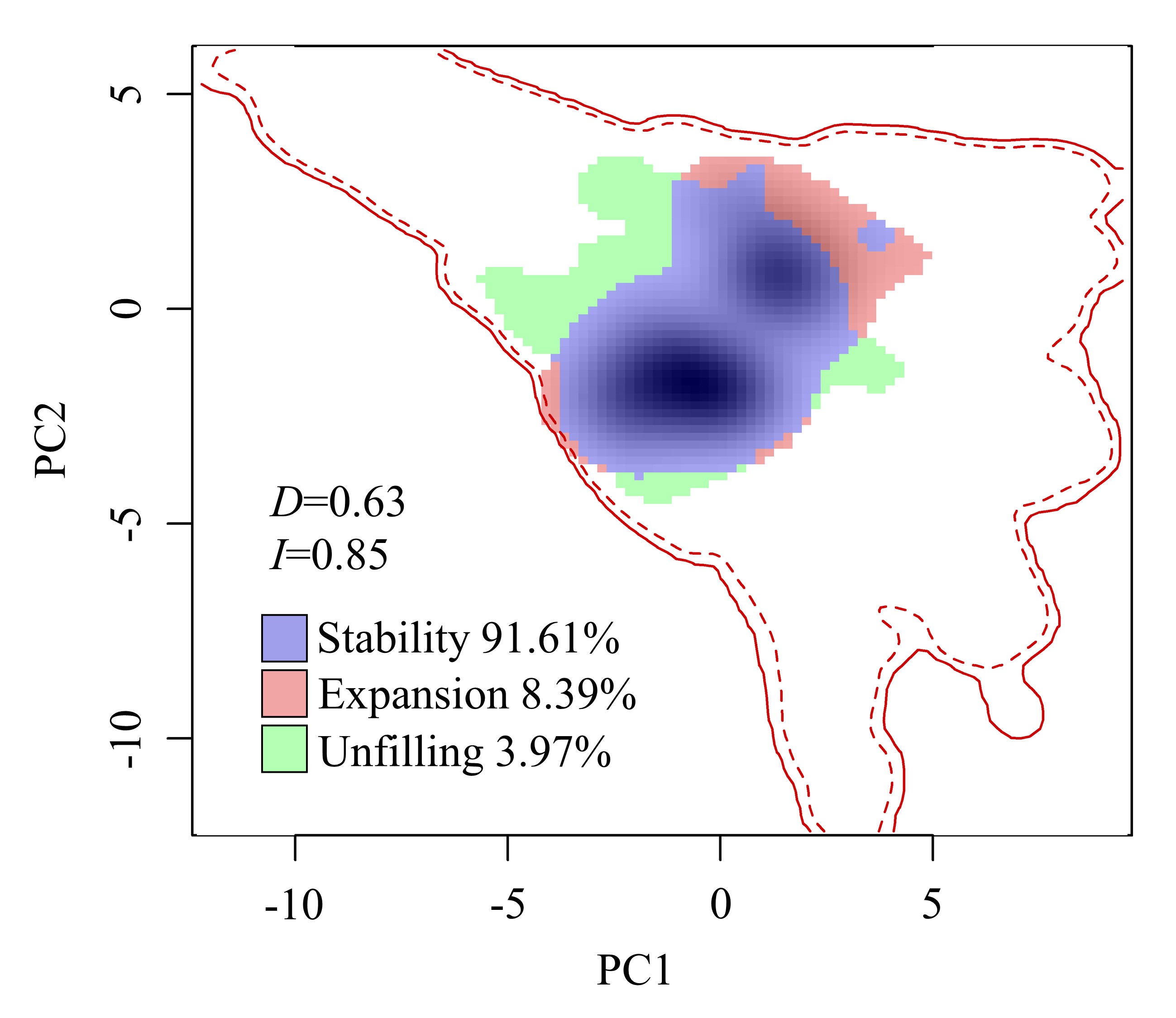

Supplement: Supplementary file 1 [file insects-15-00879-s001.zip › Fig. S3 Niche overlap between T. aureus and its hosts.jpg]

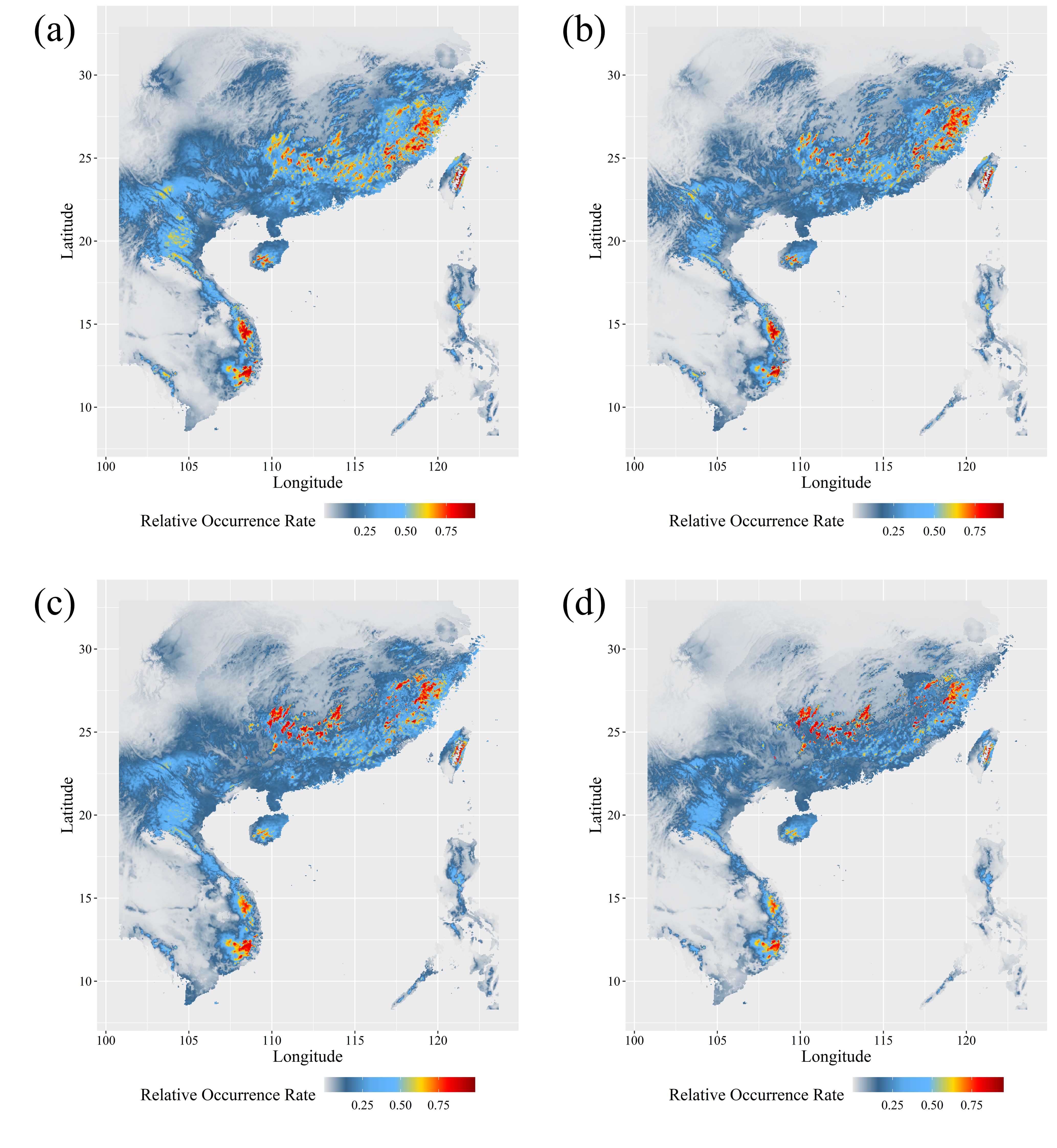

Supplement: Supplementary file 1 [file insects-15-00879-s001.zip › Fig. S4 The current potential distribution of T. aureus based on four different combinations.jpg]

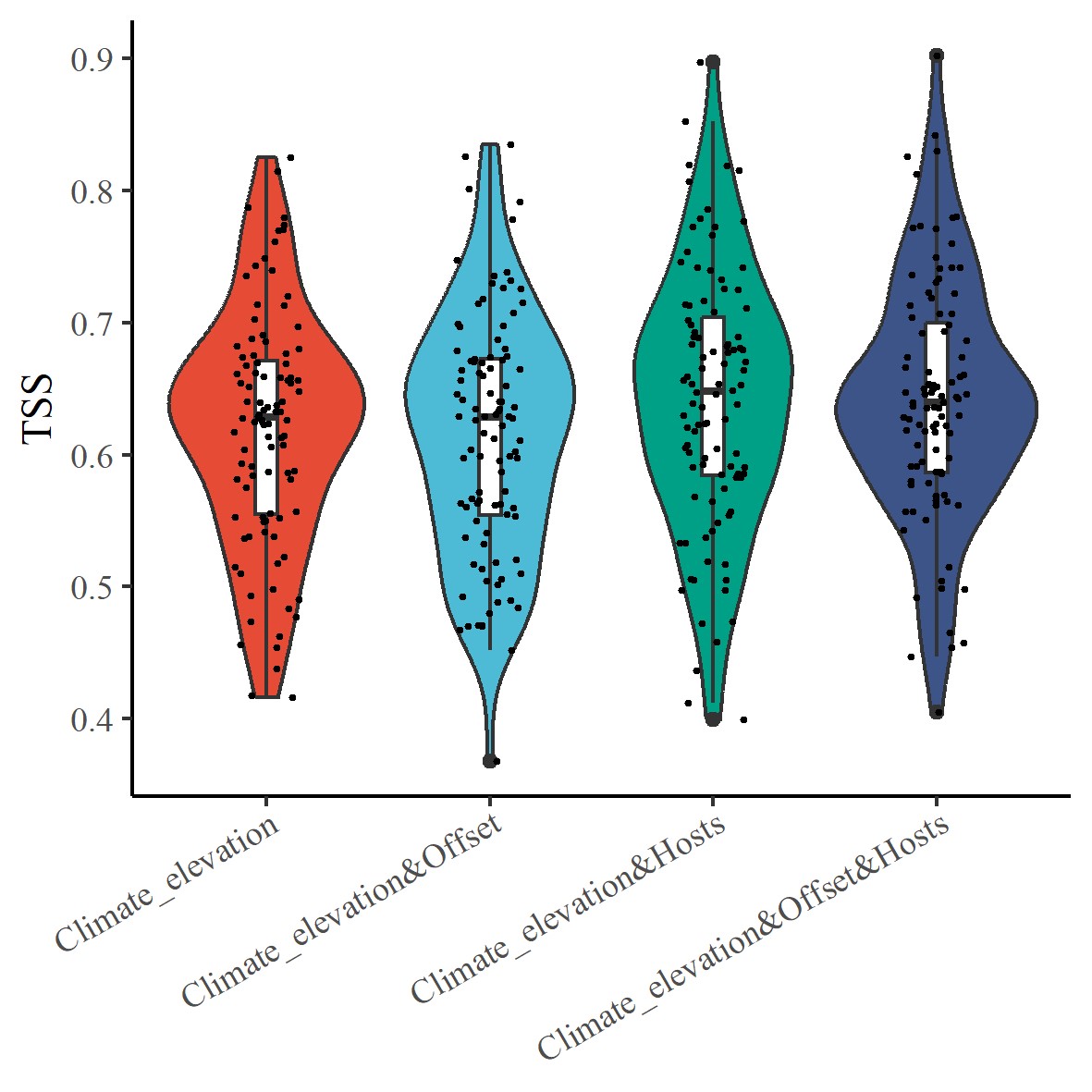

Supplement: Supplementary file 1 [file insects-15-00879-s001.zip › Fig. S5 The model performance of TSS under four different combinations of predictive variables.jpg]

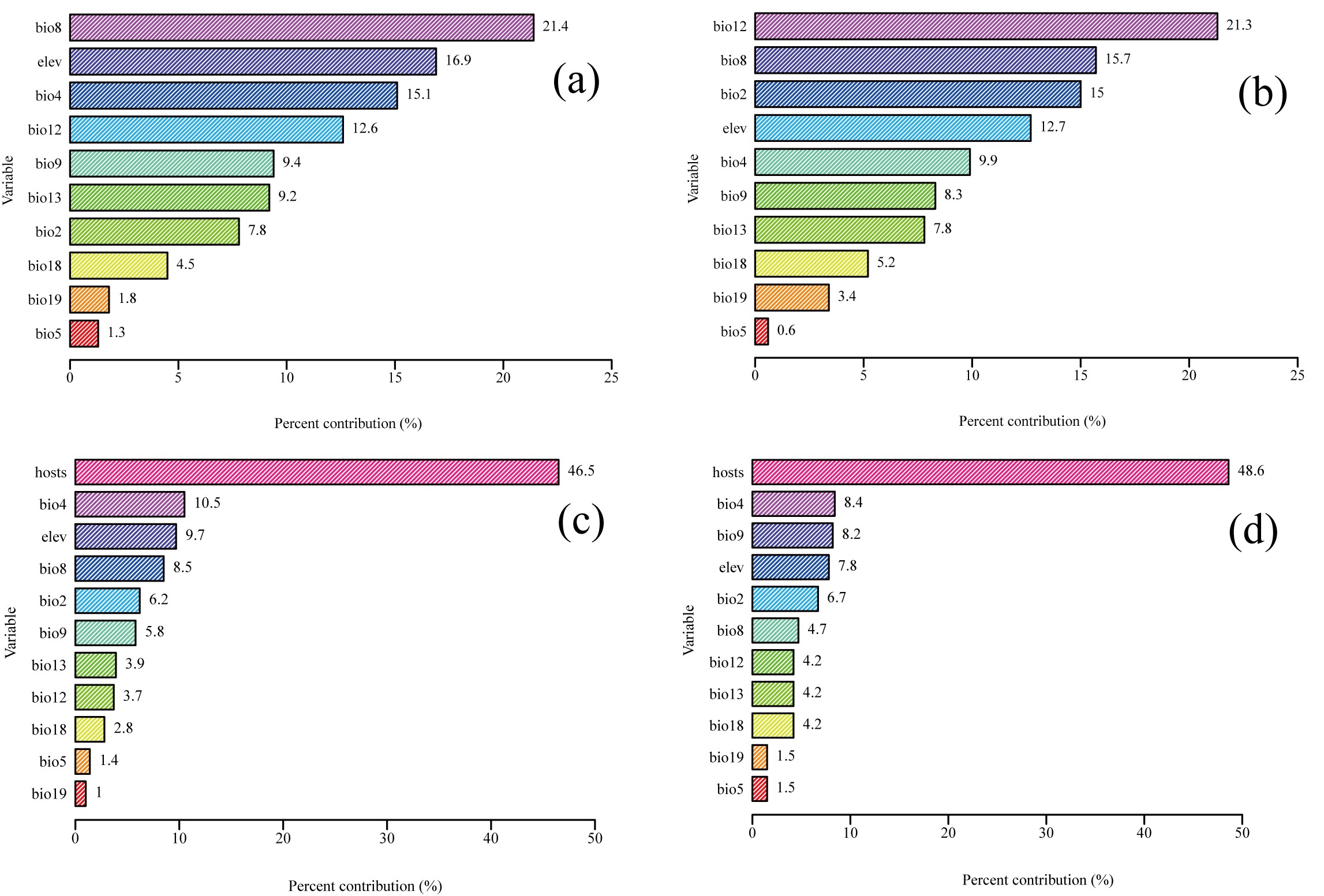

Supplement: Supplementary file 1 [file insects-15-00879-s001.zip › Fig. S6 The contribution to the current potential suitable areas of T. aureus.jpg]

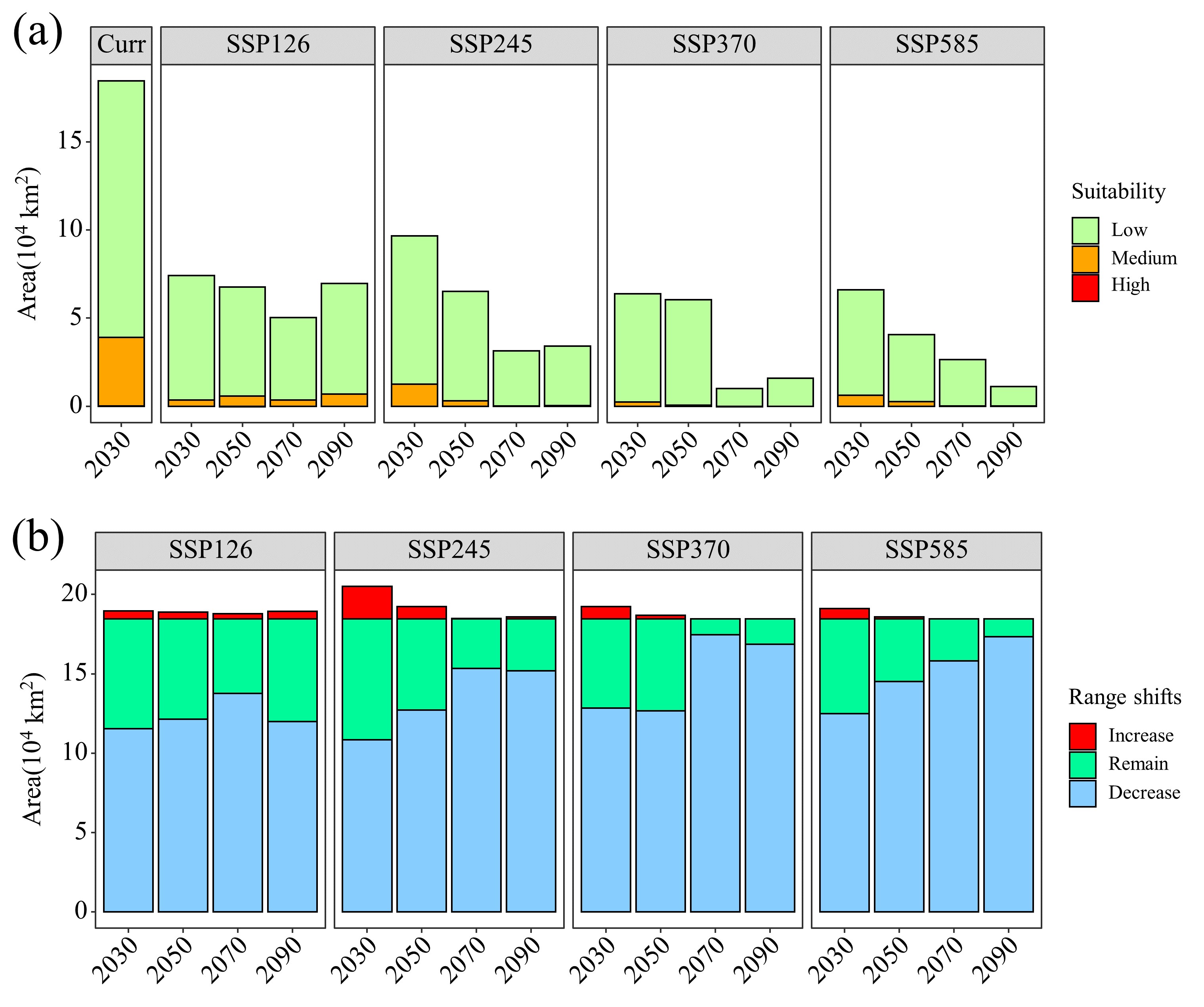

Supplement: Supplementary file 1 [file insects-15-00879-s001.zip › Fig. S7 The trend of future potential suitable areas (a) and changes (b) of T. aureus under different climate scenarios.jpg]

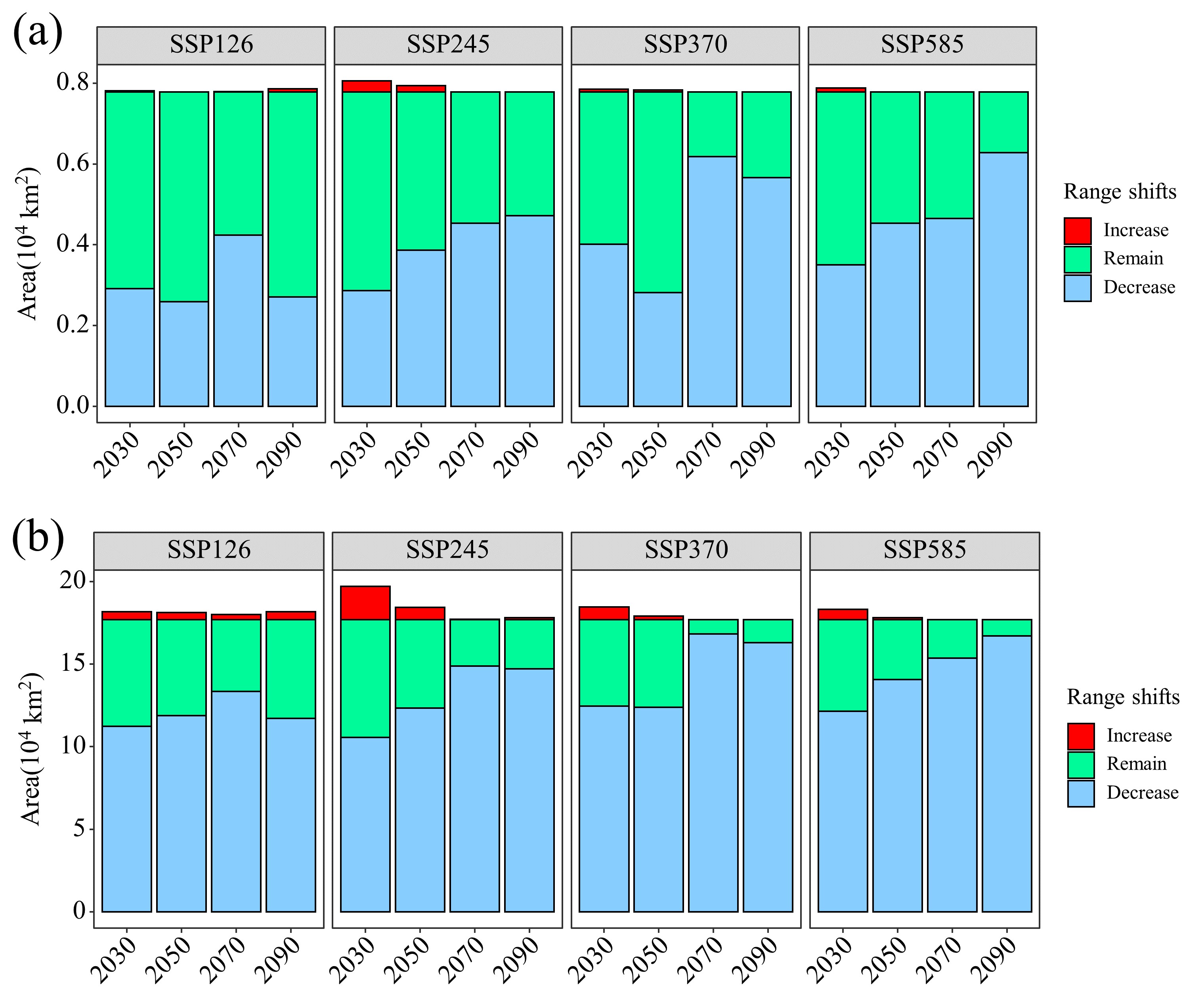

Supplement: Supplementary file 1 [file insects-15-00879-s001.zip › Fig. S8 The trend of future potential increase, stable and decrease areas (a) in and(b) out the protected zones .jpg]

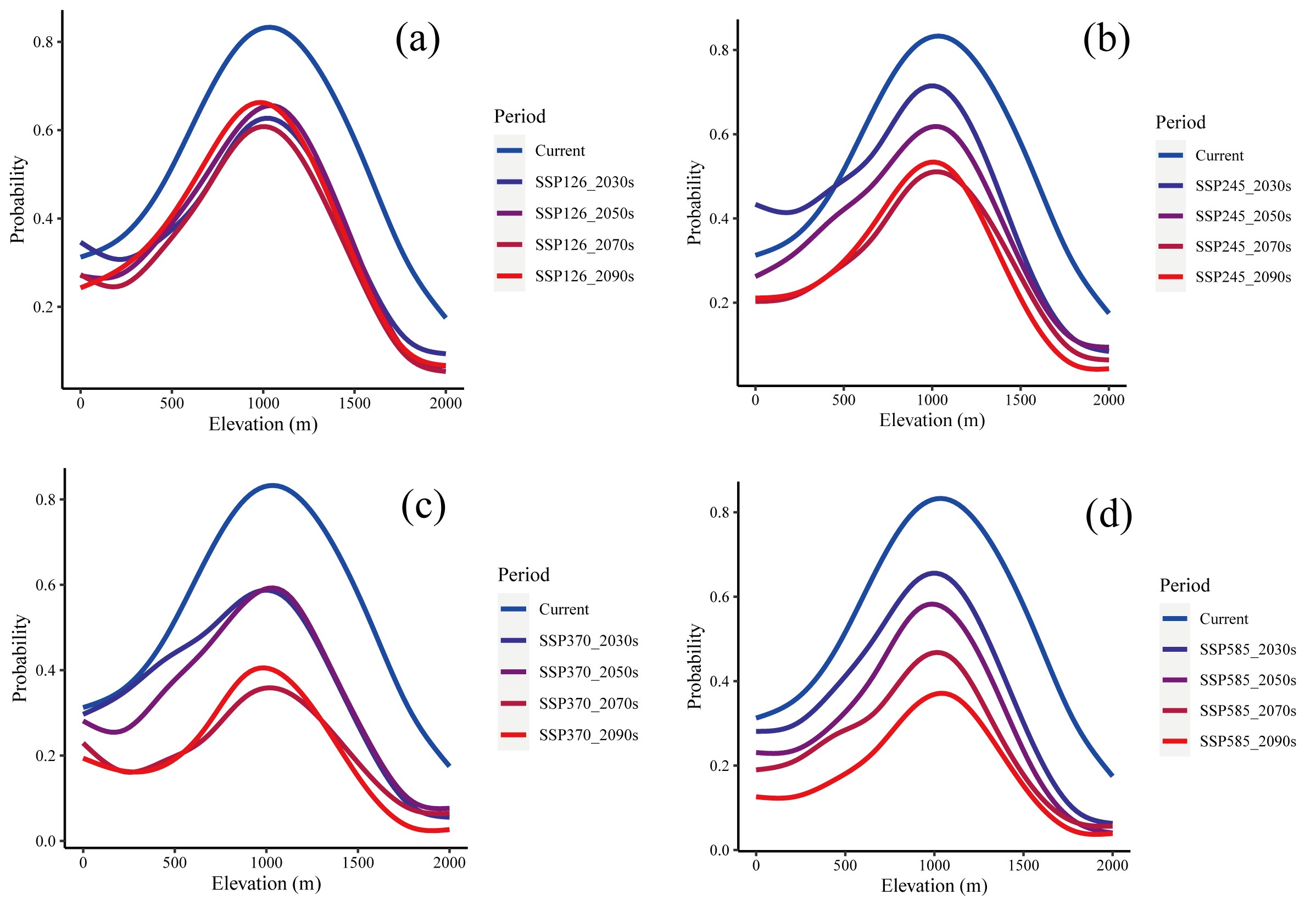

Supplement: Supplementary file 1 [file insects-15-00879-s001.zip › Fig. S9 The change in the future altitudinal niche of T. aureus.jpg]
